# Evaluating the risk for Usutu virus circulation in Europe: comparison of environmental niche models and epidemiological models

**DOI:** 10.1186/s12942-018-0155-7

**Published:** 2018-10-12

**Authors:** Yanchao Cheng, Nils Benjamin Tjaden, Anja Jaeschke, Renke Lühken, Ute Ziegler, Stephanie Margarete Thomas, Carl Beierkuhnlein

**Affiliations:** 1 Department of Biogeography, University of Bayreuth, Universitätsstr. 30, 95447 Bayreuth, Germany; 2 Bernhard Nocht Institute for Tropical Medicine, World Health Organization Collaborating Centre for Arbovirus and Hemorrhagic Fever Reference and Research, Hamburg, Germany; 3 Friedrich-Loeffler-Institut, Institute of Novel and Emerging Infectious Diseases, Südufer 10, 17493 Greifswald – Insel Riems, Germany; 4 BayCEER, Bayreuth Center for Ecology and Environmental Research, Bayreuth, Germany

**Keywords:** Usutu, Maxent, SEIR, Vector-borne disease, Risk map, Europe, Basic reproduction number, R_0_, ENM

## Abstract

**Background:**

Usutu virus (USUV) is a mosquito-borne *flavivirus*, reported in many countries of Africa and Europe, with an increasing spatial distribution and host range. Recent outbreaks leading to regional declines of European common blackbird (*Turdus merula*) populations and a rising number of human cases emphasize the need for increased awareness and spatial risk assessment.

**Methods:**

Modelling approaches in ecology and epidemiology differ substantially in their algorithms, potentially resulting in diverging model outputs. Therefore, we implemented a parallel approach incorporating two commonly applied modelling techniques: (1) Maxent, a correlation-based environmental niche model and (2) a mechanistic epidemiological susceptible-exposed-infected-removed (SEIR) model. Across Europe, surveillance data of USUV-positive birds from 2003 to 2016 was acquired to train the environmental niche model and to serve as test cases for the SEIR model. The SEIR model is mainly driven by daily mean temperature and calculates the basic reproduction number R_0_. The environmental niche model was run with long-term bio-climatic variables derived from the same source in order to estimate climatic suitability.

**Results:**

Large areas across Europe are currently suitable for USUV transmission. Both models show patterns of high risk for USUV in parts of France, in the Pannonian Basin as well as northern Italy. The environmental niche model depicts the current situation better, but with USUV still being in an invasive stage there is a chance for under-estimation of risk. Areas where transmission occurred are mostly predicted correctly by the SEIR model, but it mostly fails to resolve the temporal dynamics of USUV events. High R_0_ values predicted by the SEIR model in areas without evidence for real-life transmission suggest that it may tend towards over-estimation of risk.

**Conclusions:**

The results from our parallel-model approach highlight that relying on a single model for assessing vector-borne disease risk may lead to incomplete conclusions. Utilizing different modelling approaches is thus crucial for risk-assessment of under-studied emerging pathogens like USUV.

**Electronic supplementary material:**

The online version of this article (10.1186/s12942-018-0155-7) contains supplementary material, which is available to authorized users.

## Background

Vector-borne diseases (VBDs) are of growing importance. Due to global transport, long-distance travel, population growth, environmental and climatic changes, VBDs are emerging all over the world [1–4]. In addition to human-mediated spread, mobile species such as migratory birds are promoting long-distance transport of pathogens [5]. If the local conditions at the introduction sites (e.g. hosts, vectors, and climate) are suitable, the pathogen can establish and evolve quickly, resulting in rapid local spread [6]. Usutu virus (USUV) is an example where both processes resulted in the recent arrival and spread of a zoonotic mosquito-borne virus in Europe [5].

**Fig. 1:**
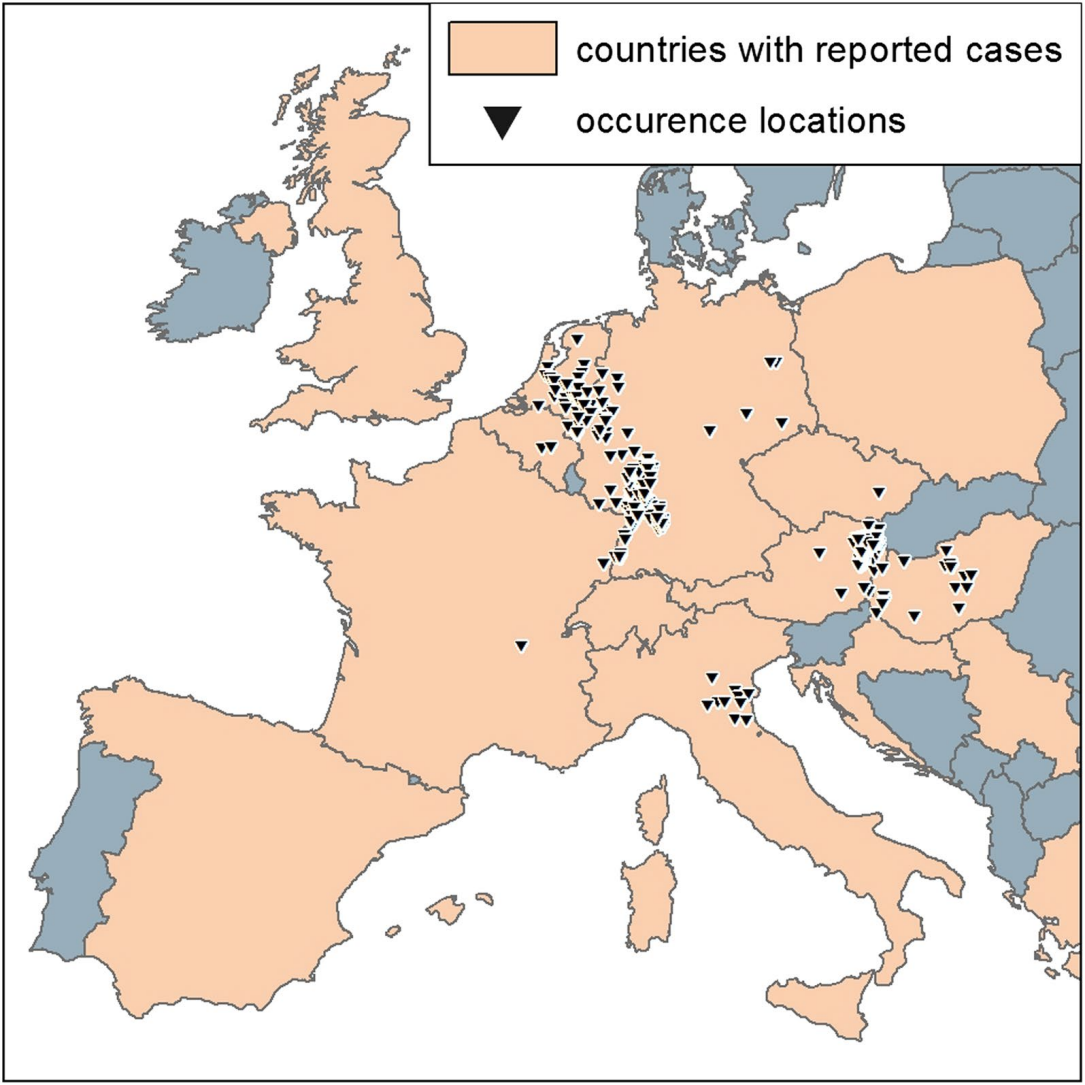
USUV in Europe. Orange areas: European countries where cases of USUV have been reported, regardless of species and method of confirmation. Triangles: Spatially explicit records of USUV occurrence 2003—2016 before spatial rarefication. These are locations where individual USUV-positive dead birds have been found, confirmed by reverse transcription polymerase chain reaction (RT-PCR)

USUV is a *flavivirus* [7] belonging to the Japanese encephalitis virus serocomplex [8]. As a member of the family Flaviviridae, USUV is a single-stranded RNA virus closely related to Murray Valley encephalitis virus, Japanese encephalitis virus, and West Nile virus (WNV) [8]. It was first isolated in 1959 from *Culex neavei* mosquitoes in Swaziland and named after the Usutu river [7]. Its most important vectors are mosquito species of the genus *Culex* [9]. Since the first record, USUV has been reported for several African countries (e.g. Senegal, Central African Republic, Nigeria, Uganda) and detected in mosquitoes, birds, and humans [10]. In Europe USUV has been detected in 15 countries, with increasing spatial distribution and host range [9, 11–15] (Fig. 1). The earliest evidence of USUV in Europe came from a dead common blackbird (*Turdus merula*) found in Italy in 1996, although this case was not identified as such until 2013 [16]. The first USUV epidemic in Europe was a series of dead common blackbirds reported from Austria in 2001 [17]. In the subsequent years, USUV was reported in further European countries. USUV or corresponding antibodies were detected in horses, bats, dogs [11, 18, 19], and at least 58 bird species, with common blackbirds as dominant avian host [14].

In 2009, the first human case of USUV infection in Europe was reported in Italy [20], followed by further human cases in Germany [21, 22], Croatia [23], Austria [24], and France [25]. Human cases are commonly characterized by mild symptoms including fever, rash, jaundice, headache, nuchal rigidity, hand tremor and hyperreflexia [20, 23, 26, 27]. However, at least in immunosuppressed patients USUV can cause a neuro-invasive infection [20], and it has recently been suspected to have caused idiopathic facial paralysis [25]. In addition to that, USUV infections were also detected from blood donors and healthy forestry workers in Germany and Italy [21, 22, 28], suggesting that asymptomatic infections can occur among humans. Recent data from Italy indicate that human USUV infections may not be a sporadic event and can even be more frequent than WNV infections in areas where both viruses co-circulate [9, 29, 30]. Furthermore, due to cross reactions in antibody tests, the number of human USUV cases may be underestimated through confusion with other flaviviruses [26]. As a consequence, the actual distribution of USUV and associated number of cases is likely to be larger than currently known [31].

The transmission cycle with birds as enzootic hosts creates a complex setting related to the risk for human health. First, migratory birds may transport the pathogen over large distances and can cause repeated re-introduction of the virus into a specific region that is not appropriate to maintain an outlasting population of the pathogen [5]. Second, common blackbirds are the predominant host [9, 14]. This species is very common across Europe and has grown accustomed to urban habitats, exhibiting high population densities in human settlements [32]. This means that vectors only need to cover short spatial distances between infected birds and humans—and the widespread mosquito species *Cx. pipiens* is a known bridge vector between mammals, birds and humans [33, 34]. In consequence, USUV is becoming an increasing threat for Europe as a mosquito-borne and zoonotic disease. Measures should be undertaken to improve or even create awareness towards zoonotic VBDs. For this purpose, spatial representations of risk are needed.

Models for vector borne viral diseases can be generated at various spatial and temporal scales [35]. Maps of vector occurrence or disease transmission risk derived from them can be used to direct vector surveillance and control programs as well as to inform public health officials, medicine practitioners and the general public about potential risks. Current approaches can be divided into two basic groups: correlative models (e.g. environmental niche models) and process-based models (e.g. epidemiological models). Both types of models have their own strengths and weaknesses [35]. Correlative environmental niche models, on the one hand, typically utilize species occurrence records and environmental predictor variables to estimate the current and future potential spatial distribution of a target species [36] or disease [37–42]. They do not require a priori knowledge about the specific effects single variables have, and are typically used on coarser spatio-temporal scales [35]. Process-based epidemiological models, on the other hand, aim to simulate the entire transmission process. Using knowledge gained from laboratory experiments or field observations, they require a deeper understanding of disease dynamics. As all models for VBD have their individual strengths and weaknesses, it is best practice not to rely on a single approach, but draw a conclusion from a consensus of multiple different models [35]. Although both model categories are widely used when modeling VBDs [35], comparisons of different models’ outputs are typically made within those categories (e.g. [43]), and a comparison across categories is still missing.

To date only a limited number of USUV models for spatially confined areas exist. Based on an epidemiological model for WNV, Rubel et al. [44] developed a mechanistic susceptible-exposed-infected-removed (SEIR) model for USUV in Vienna (Austria) [44–46], which was later successfully applied to Germany and neighboring countries [47]. This model is mainly driven by daily mean temperature, and to enable the comparison of modeled bird deaths and observed bird deaths, it was originally carried out with interpolated monthly mean temperature values so as to achieve the same temporal resolution as the available bird death data [44]. A different, environmental niche model-based approach was followed by Lühken et al. [31], who adopted boosted regression trees to assess the spatio-temporal risk for USUV in Germany by estimating the risk in each grid cell.

Here we present, for the first time, USUV risk maps covering the entirety of the European mainland. Using two models in parallel, we utilize the mechanistic SEIR model by Rubel et al. [44] as well as a newly developed environmental niche model based on the machine-learning technique Maxent. Instead of using interpolated monthly mean temperature values for a single location, rasterized daily mean temperature was used to run the SEIR model. In order to increase comparability between the models, the same data source was also applied for the use of Maxent. Spatial risk maps were generated by both models. By using models from these two different groups, we are aiming at (1) estimating the potential risk for USUV transmission under current climate conditions in Europe and (2) investigating the differences between the outputs of two widely-used modelling approaches, which could be a first step towards interdisciplinary model comparison.

## Methods

### Study area and USUV occurrence records

In this study, we focus on current European occurrence records of USUV in the years of 2003–2016, from the earliest to the latest USUV cases available. The investigation area is limited by the natural coastlines, as well as through the reported USUV locations in Eastern Europe (Fig. 1).

To achieve a good data quality, only locations of USUV-positive birds confirmed by reverse transcription polymerase chain reaction (RT- PCR) were taken into account. This was done because (1) data from USUV-positive mammals or mosquitoes are collected quite unsystematic, i.e. data on USUV-positive birds are most consistent and comparable between the different European countries, and (2) other methods such as antibody analysis may not be able to distinguish USUV from other closely related flaviviruses such as WNV [48]. According to this rule, a total number of 376 USUV records was collected. USUV-positive data in Germany were collected by the German Mosquito Control Association (KABS), the Nature and Biodiversity Conservation Union (NABU), the local veterinary authorities and/or by the local state veterinary laboratories [47, 49–51]. Records for other European countries were derived from the literature (Additional file 1): Geographical coordinates published in the literature were directly entered into the database, precise site descriptions were digitized using Google Earth Pro, and high-quality occurrence maps were geo-referenced using ESRI ArcGIS 10.2.2.

### Climate data

Time series of daily mean temperature data, required by the SEIR model, were acquired from the E-OBS dataset version 15.0 [52] on a regular latitude–longitude grid with a spatial resolution of 0.25° (about 20 km). E-OBS provides gridded daily temperature and precipitation data for Europe based on data from weather stations. To compare the results from the SEIR model and the environmental niche model properly, bio-climatic variables, which are required by the environmental niche model, were generated from the E-OBS dataset as well. Therefore, time series of daily minimum, maximum temperature and daily precipitation sums were acquired in addition to daily mean temperature.

Since the occurrence records for USUV cover the years of 2003–2016, these time series were trimmed accordingly. Considering that the spatial coverage of the E-OBS time series varies over time, grid cells with more than 10% missing data were excluded from our analyses. Monthly mean values were derived using the “raster” package [53] for R 3.2.1 [54] and 19 bio-climatic variables were calculated in SAGA-GIS version 2.1.4 [55] for use with the environmental niche model.

### Environmental niche model: Maxent

For the environmental niche model, we used Maxent 3.3.3k [56]. Maxent is a powerful machine-learning technique that is widely used [35] to model the potential distribution of species, especially when the occurrence data are sparse [57]. Using occurrence records and environmental predictor variables as input data, Maxent generates maps of environmental suitability for transmission of USUV. Ranging between 0 for the lowest and 1 for the highest suitability, these maps can optionally be converted into presence/absence maps by applying a threshold value.

**Fig. 2:**
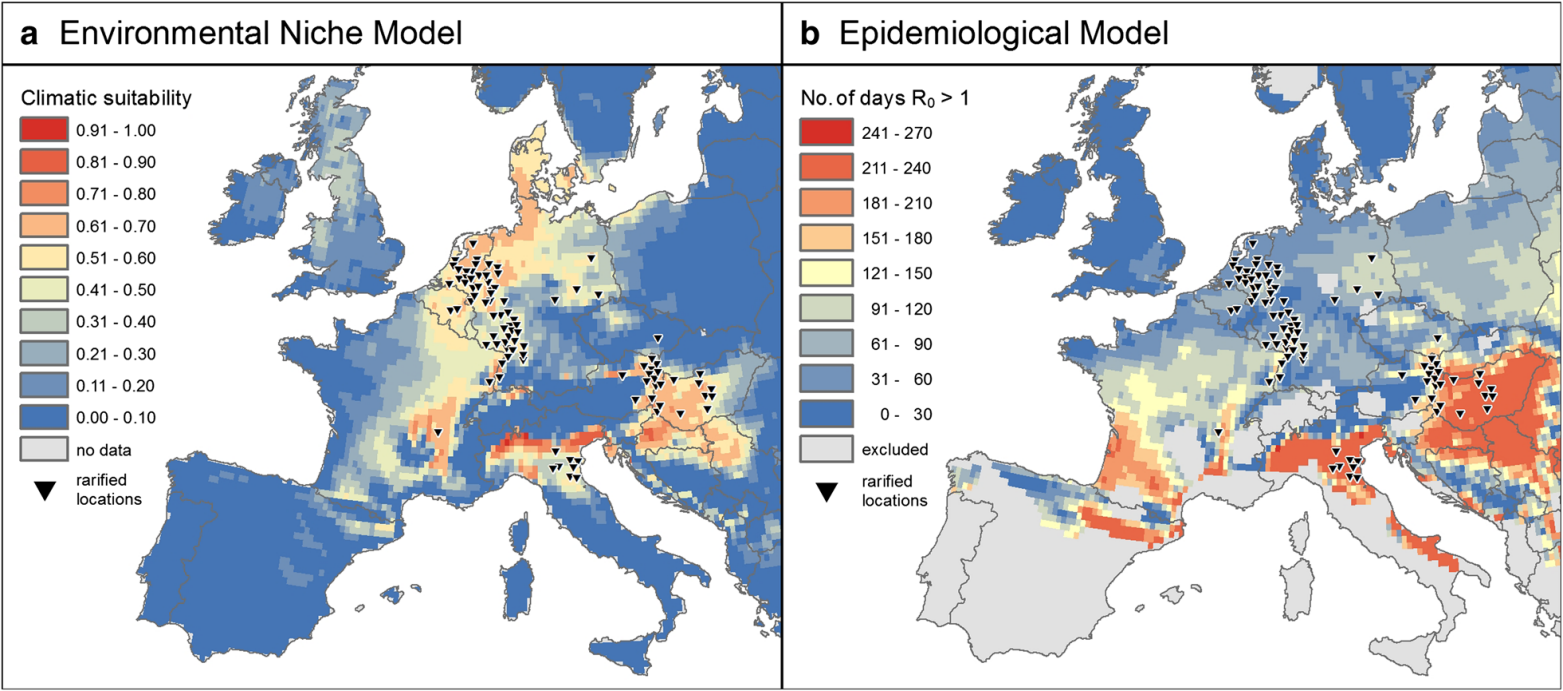
Potential geographic distribution of USUV in Europe. **a** Climatic suitability estimated by the environmental niche model, and **b** the yearly mean absolute number of days of R_0_ > 1 simulated by the epidemiological SEIR model. Gray areas in **b** denote regions with a dry season that were not included in the SEIR model. Both models use the same E-OBS climate data for 2003–2016. Locations of recorded cases for the environmental niche model were rarified (in comparison to Fig. 1) to avoid spatial autocorrelation (see “Methods”)

Maxent models are fitted assuming that all locations in the landscape are equally likely to be sampled. However, when the occurrence records are collected with different methods, sampling bias is inevitable. Compared to other methods, systematic sampling, also called spatial filtering of biased records [58], has a good performance regardless of species and bias type [58, 59]. It was applied by using the *SDM tool box* [60], an addon for ESRI ArcGIS that provides advanced tools and convenience functions for the Maxent workflow. To determine an appropriate spatial filtering resolution (the minimum distance between any two locations), the following rules were taken into consideration: (1) The spatial filtering process should decrease the bias distribution, but the remaining records should still represent the observed spatial patterns well. (2) There should be enough records left to run Maxent after spatial filtering. Consequently, the spatial filtering resolution was set to 20 km (about 0.25°), and 92 USUV records left after filtering in order to achieve optimum results and to avoid artefacts (Fig. 2).

Selection of the environmental predictors for the model followed a two-step approach (Table 1). First, 8 out of the 19 bio-climatic variables that were deemed unsuitable for the task were excluded due to the following ecological reasons: BIO2 and 3 (“mean diurnal range” and “isothermality”) were excluded because while daily fluctuations in temperature are important for the mosquito life cycle and transmission dynamics, the monthly averages available here were considered unsuitable for capturing such short-term fluctuations. BIO12 (“annual precipitation”) was excluded because summer and winter precipitation play very different roles in this context and should be considered separately. All variables referring to the wettest/driest quarter or month of the year (BIO8, 9, 13, 14, 16, and 17) were excluded because seasonal precipitation patterns vary largely across Europe. As such, the wettest time of the year can be summer in some regions and winter in others, making this kind of variable unsuitable for larger scale analyses. The remaining eleven variables were further reduced through the built-in Jackknife feature in Maxent with a ten-fold cross-validation run, following the recommendations of Elith et al. [61]. In the end, a combination of five variables was chosen, consisting of annual mean temperature, minimum temperature of coldest month, mean temperature of coldest quarter, precipitation seasonality, and precipitation of warmest quarter. We used default settings for Maxent (10,000 background locations, 500 iterations), but disabled the use of “threshold” and “hinge” features, that would have led to over-fitting due to an inappropriate amount of model complexity.

**Table 1:** Excluded and selected environmental predictor variables for the environmental niche model

Abbreviation	Variables
Excluded—monthly minima and maxima are not suitable to estimate daily fluctuations	
BIO2	Mean diurnal range (mean of monthly (max temp − min temp))
BIO3	Isothermality (BIO2/BIO7) × 100
Excluded—summer and winter precipitation are important to distinguish for mosquitoes and disease transmission dynamics	
BIO12	Annual precipitation
Excluded—wettest/driest time of the year can be in different seasons across Europe	
BIO8	Mean temperature of wettest quarter
BIO9	Mean temperature of driest quarter
BIO13	Precipitation of wettest month
BIO14	Precipitation of driest month
BIO16	Precipitation of wettest quarter
BIO17	Precipitation of driest quarter
Excluded by Jackknife	
BIO4	Temperature seasonality (standard deviation × 100)
BIO5	Maximum temperature of warmest month
BIO7	Temperature annual range (BIO5–BIO6)
BIO10	Mean temperature of warmest quarter
BIO19	Precipitation of coldest quarter
Model input	
BIO1	Annual mean temperature
BIO6	Minimum temperature of coldest month
BIO11	Mean temperature of coldest quarter
BIO15	Precipitation seasonality (coefficient of variation)
BIO18	Precipitation of warmest quarter

Maxent, like many other environmental niche model approaches, generates pseudo-absence (“background”) locations to make up for the lack of field records of true absence of the target species. Careful selection of the area from which these background locations are allowed to be drawn from is an important part of model creation, as it can affect model performance and results. According to Barve et al. [62], this should be done by requiring the background locations to be within the area the species could realistically disperse to. We followed a buffer-based method [63] by setting a series of buffer radii from 0.5° to 24° (see Additional file 2), given the grid cell size of 0.25°. It is suggested to take the radius when the model performance stops increasing [63]. In addition to the built-in AUC (area under the receiver operator characteristic curve), true skill statistic (TSS) was also calculated as an indicator of model performance (Additional file 2). A radius of 12° was chosen as suggested, with the final model reaching an AUC of 0.92 and a TSS score of 0.78, both suggesting good model performance. In this model, the minimum temperature of the coldest month had the strongest contribution to the model (58%), followed by precipitation of the warmest quarter (21%) and annual mean temperature (13%). The threshold for distinguishing predicted presence and absence was based on the receiver operator characteristic (ROC), choosing the point along the ROC curve that maximized the sum of sensitivity and specificity. We chose this criterion also known as “maxSSS” because it is objective [64], widely used, performs consistently well with presence-only data [65, 66] and delivers threshold values that are relatively low [66], facilitating the high sensitivity desired in risk assessment studies.

### Epidemiological model: SEIR

The SEIR model used in this study was developed by Rubel et al. [44] for Vienna (Austria) and surrounding areas based on data from different parts of the world. The model simulates the seasonal life cycles and inter-species USUV infections of the main vector and host species, *Cx. pipiens* and *T. merula* respectively. Health states of birds and mosquitoes are classified into nine compartments (larvae state of mosquitoes, health states susceptible/latent infected/infectious of mosquitoes and birds as well as recovered and dead birds, see [44]), and described by ordinary differential equations (see Additional file 3). The basic reproduction number R_0_ is then calculated as the dominant eigenvalue of the next-generation matrix as described in [67], resulting in (see Table 2 for model parameters and Additional file 3 for details):

$$R_{0} = \sqrt {\left[ {\frac{{\delta_{M} \gamma_{M} \beta_{M} }}{{\left( {\gamma_{M} + m_{M} } \right)m_{M} }}\frac{{S_{B} }}{{K_{B} }}} \right]\left[ {\frac{{\delta_{M} \gamma_{B} \beta_{B} }}{{\left( {\gamma_{B} + m_{B} } \right)\left( {\alpha_{B} + m_{B} } \right)}}\frac{{S_{M} }}{{K_{B} }}} \right]}$$

The SEIR model is mainly driven by variables responding to temperature. Further drivers are latitude, calendar day, and parameters with constant values [44].

**Table 2:** Variables and parameters in the R_0_ equation, following [44]

Parameter		Value
Mosquitoes		
Mortality rate	$m_{M}$	$m_{M} \left( T \right) = 0.00025T^{2} - 0.0094T + 0.10257$
		$T$: daily mean temperature
Biting rate	$\kappa$	$\kappa \left( T \right) = \frac{0.344}{{1 + 1.231{ \exp }\left( { - 0.184\left( {T - 20} \right)} \right)}}$
Product of biting rate ($\kappa$) and transmission possibility from mosquitoes to birds ($P_{M}$)	$\beta_{M}$	$\beta_{M} \left( T \right) = P_{M} \kappa \left( T \right)$ $P_{M}$ = 1
Percentage of non-hibernating mosquitoes	$\delta_{M}$	$\delta_{M} = 1 - \frac{1}{{1 + 1775.7{ \exp }\left[ {1.559\left( {D - 18.177} \right)} \right]}}$
		$$D = 7.639arcsin\left[ {{ \tan }\left( \epsilon \right){ \tan }\left( \varphi \right) + \frac{0.0146}{{{ \cos }\left( \epsilon \right){ \cos }\left( \varphi \right)}}} \right] + 12$$
		$$\epsilon = 0.409sin\left( {\frac{{2\pi \left( {d - 80} \right)}}{365}} \right)$$
		$D$: daytime length,$\epsilon$: declination, $\varphi$: geographic latitude
Exposed—infected/infectious rate	$\gamma_{M}$	$\gamma_{M} \left( T \right) = 0.0093T - 0.1352$, $T \ge 15^{^\circ }$ $\gamma_{M} \left( T \right) = 0$, $T < 15^{^\circ }$
Susceptible mosquito population	$S_{M}$	Dynamic value, see Additional file 3
Birds		
Mortality rate	$m_{B}$	0.0012
Removal rate: fraction of infected birds either recovering or dying	$\alpha_{B}$	0.182
Exposed—infected/infectious rate	$\gamma_{B}$	0.667
Product of biting rate ($\kappa$) and transmission possibility from birds to mosquitoes ($P_{B}$)	$\beta_{B}$	$\beta_{B} \left( T \right) = P_{B} \kappa \left( T \right)$ $P_{B}$ = $0.125$
Susceptible black bird population	$S_{B}$	Dynamic value, see Additional file 3
Environmental capacity	$K_{B}$	see Additional file 3

The original SEIR R-code of the model was upgraded to work on a spatial grid rather than a single point location, and daytime length was calculated for each grid cell based on the geographical latitude of its center. Instead of interpolating daily data from monthly mean temperature, the model was run with true daily temperature data from the E-OBS dataset [52]. As an extensive literature review did not yield any new information, all other variables and parameters originally used by Rubel et al. were maintained in this study.

As the SEIR model for USUV was created for and calibrated within a temperate climate, water availability or precipitation were not considered a limiting factor by the developers. However, this assumption is not applicable for the entire study area, as the dry summers of Mediterranean climates can lead to a different, two peaked activity pattern of *Cx. pipiens* mosquitoes [68]. Consequently, the model was applied only to regions with a climate that is classified as cold or temperate with warm to hot summers but no dry season (Cfa, Cfb, Dfa and Dfb in the Köppen-Geiger system [69, 70]) (Fig. 2b).

The basic reproduction number R_0_ (the number of secondary cases arising from a single infection in an otherwise uninfected population) of USUV calculated by the SEIR model is a threshold value: if R_0_ > 1, an outbreak is possible after a single introduction of the pathogen; whereas if R_0_ < 1, the introduced virus population will die out [67]. The daily R_0_ value of each cell within the spatial raster was calculated within the time span of 2003-01-01 to 2016-12-31. From this, the average yearly number of days with R_0_ > 1 was calculated for each raster cell and the maxSSS threshold was calculated for direct comparison with the environmental niche model based on the same presence and background locations that were used in the Maxent model. In addition to that, the average daily R_0_ value of the main transmission season (June–September) was calculated for each year and raster cell.

## Results

**Fig. 3:**
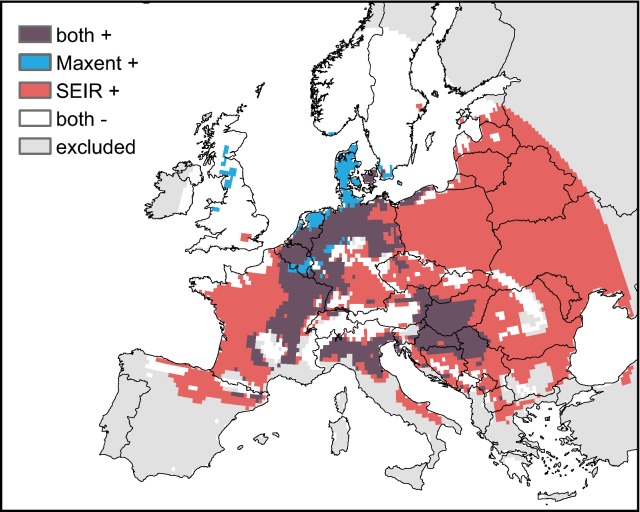
Areas of agreement and disagreement of both models. Dark purple areas denote regions where both models predict suitable conditions for USUV-transmission based on the maxSSS threshold. In the blue and red areas, only the environmental niche model and SEIR predict suitable conditions, respectively. In white areas none of the models predicts suitable environmental conditions, while gray areas were excluded from further analyses because they are outside the climatic zones the SEIR model was developed for, or outside the buffer applied to the Maxent model

The potential geographic distribution of USUV predicted by both models on the continental European scale are shown in continuous form in Fig. 2, and as a direct comparison based on the maxSSS thresholds (environmental niche model: 0.35 in Maxent’s logistic output format, epidemiological model: 40 days of R_0_ > 1) in Fig. 3. While there are differences between the two models in parts of the study area, 15% of the study area are projected to be suitable by both approaches. The northern Italian outbreak region in and around the Po Valley is identified as a highly suitable area for USUV by both models. The same is true for eastern Austria, the Pannonian Basin and adjoining areas, as well as a narrow strip along the Rhône river in France. Large parts of north-eastern France, the Benelux states and western and northern Germany are predicted to be at least somewhat suitable by both models. On the other hand, environmental niche model and SEIR agree on low risk being present in northern and mountainous regions (such as Sweden, Norway and the British Isles), where relatively low average and minimum temperatures keep the probability of transmission low.

In general, the environmental niche model accurately determines the occurrences of birds found positive with USUV. Compared to the SEIR, it suggests elevated climatic suitability for USUV to the north and west of the Jura Mountains as well as northwards along the Rhine and the North Sea coast until southern Denmark (Fig. 2a). Following the maxSSS threshold, the environmental niche model predicts a total of 17% of the study area to be suitable for transmission (sensitivity: 0.946, specificity: 0.852). 2% of the entire area are considered suitable only by the environmental niche model and not by the SEIR, including most parts of Denmark and adjoining parts of northern Germany, northern Netherlands, southern Belgium and a few areas in northern Britain (Fig. 3).

In contrast, the average yearly number of days with R_0_ > 1 derived from the SEIR suggests a high risk for USUV in southwestern France and southeastern Italy, but shows relatively low risk in the northern Germany-Netherlands-Belgium region (Fig. 2b). North of the Pyrenees, the former French regions of Aquitaine and Midi-Pyrénées show a high transmission potential as well. Medium values mainly occur in Poland and northeastern Germany, along the Upper Rhine Valley and in central France. For the outbreak area in the Netherlands and northern Germany, the SEIR in this form suggests relatively low risk of transmission. However, following the maxSSS threshold, most of this region can still be classified as suitable for USUV transmission (Fig. 3). A total of 67% of the whole study area lies above the threshold for this model, resulting in a sensitivity that is slightly higher (0.989) than that of the environmental niche model but a very low specificity (0.274).

**Fig. 4:**
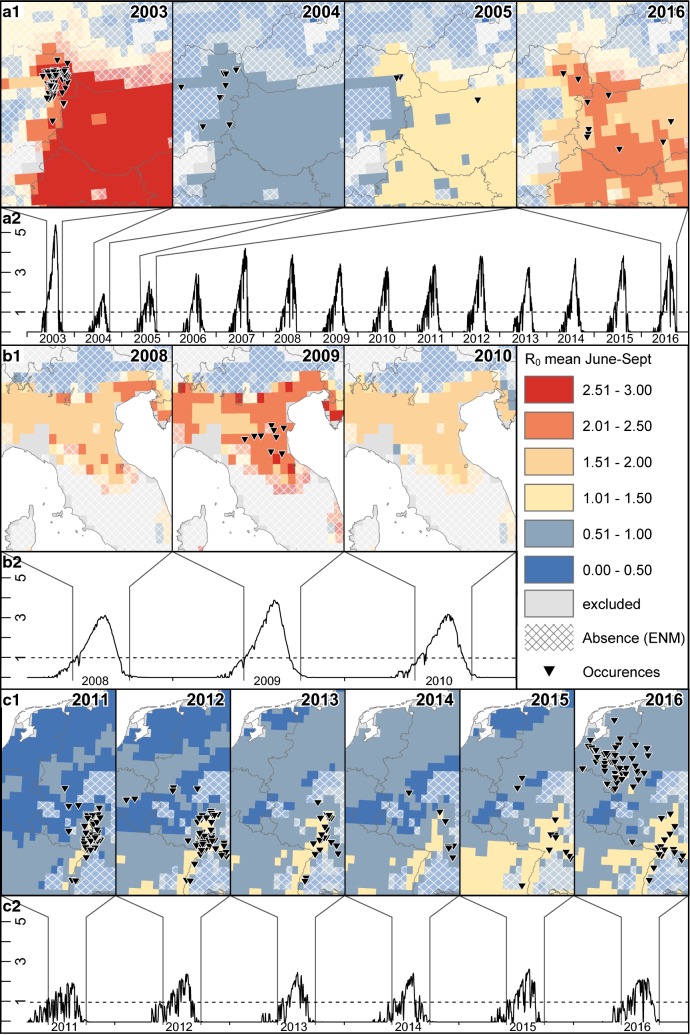
Temporal patterns of the average R_0_ values for three selected regions of Europe. **a** Austria and the Pannonian Basin, **b** northern Italy, and **c** Germany and the Netherlands. (1) Spatial representation of both models for years with USUV events. Color coding in the maps shows the average daily R_0_ values throughout June to September for the given years. Gray areas denote climate types with dry seasons, thus the SEIR model was not applied there. Cross-hatching indicates areas where the environmental niche model suggests absence of USUV, based on climate data for the whole time period from 2003 to 2016. (2) Time series curves illustrate the daily R_0_ value, averaged over all occurrence records of the respective region for each given year

Zooming in towards the main areas of observed USUV transmission allows a closer inspection of the models. In the Austrian-Hungarian outbreak area, Maxent predicts climatic suitability values sufficient for USUV transmission at all observed occurrences (Fig. 4a1). The SEIR model predicts the highest R_0_ values for the largest USUV event in 2003 (Fig. 4a2) and considerably lower values for the following 2 years with less observed cases (Fig. 4a). Relatively high R_0_ values are observed again for the last USUV event in 2016. Interestingly, though, values for the USUV-free years of 2006–2015 are higher than those of 2004/5 (Fig. 4a2).

In Italy, Maxent is able to predict the general outbreak area (Fig. 4b1). The SEIR model predicts elevated R_0_ values for the year of 2009 where USUV occurred, but similarly high values for the USUV-free years before and after (Fig. 4b2).

In the largest outbreak area in western Germany and the Benelux states, Maxent closely resembles the observed pattern of USUV occurrence (Fig. 4c1). Compared to the other two regions, the SEIR model in these areas shows much lower average and absolute R_0_ values as well as higher temporal variability throughout the transmission season (Fig. 4c2). Average R_0_ values for the transmission season rise above 1 and match the occurrence records well in the Rhine Valley but stay below 1 in the northern parts of the area, i.e. the Netherlands and northwestern Germany.

## Discussion

In face of emerging VBDs and rapid spread into new regions with suitable climatic conditions, models that show the current geographic regions at risk are required to allow local health authorities to be prepared. However, modelling approaches can differ substantially in philosophy, structure, and algorithms. Pros and cons of different approaches are evident and, obviously, there is not one single approach to be preferred for every pathogen, area or timespan.

In this study, two fundamentally different models were applied to describe the current emergence of USUV in Europe. This disease exhibits a series of complex interactions between the virus, vectors and host species [9]. Process-based models offer direct links between model outcome and underlying mechanisms, which makes interpretation of the observed spatial patterns relatively straightforward. However, exact knowledge on the parameters of USUV transmission is still scarce. With large numbers of USUV-positive birds reported from distinct geographical hot spots, the application of biogeographical distribution models may be a viable alternative. In order to identify coinciding and deviating model output, we ran the analyses based on the same climate data and following standard processes to detect regions at risk for the transmission of USUV.

The large-scale spatial patterns predicted by the two models (Figs. 2, 3) are quite similar close to the observed USUV events—with the notable exception of northern Germany and the Netherlands. Here, the environmental niche model favors higher latitudes as far north as Denmark, while the epidemiological model suggests good conditions for transmission in southwestern France and northeastern Spain (Fig. 2b) and at least suitable conditions for most parts of Eastern Europe (Fig. 3). Given the observed recent increase in temperatures across Europe and the projected further increase during the upcoming century [IPCC] [71], it can be expected that both models under-estimate future potential for USUV transmission to some degree. If precipitation patterns change dramatically so as to affect mosquito populations, the SEIR model may not be a reliable option any more in some regions. Similarly, both models are not suitable to predict today’s potential for USUV transmission in areas that are climatically very different from the study region.

### Environmental niche model

As the environmental niche model is strongly driven by existing spatial records, it is not surprising that it reflects the current distribution of USUV records better. However, it has to be kept in mind that there is no consistent monitoring of USUV across Europe, leading to biases in the occurrence records. For instance, many USUV events were reported in Italy, Austria, Hungary, and Croatia (though no RT-PCR positive birds), but to date no USUV case was reported in their neighbor countries—Slovenia and Slovakia. Due to the same reason, only bird cases were included in our approach, as it is the least biased dataset in Europe, compared to USUV cases from wild mammals (e.g. bats and wild boars) or humans. Furthermore, we restricted our USUV dataset to USUV cases confirmed by RT-PCR counts, as other methods bear the possibility of false positives that would lead to overestimation of risk. Given the high activity of West Nile Virus in the area that could easily be mistaken for USUV in antibody tests, the gain from avoiding false positives should outweigh the loss from potentially excluding some true positives. Even though Maxent is relatively insensitive to sampling bias compared to other environmental niche models [57] and records were spatially rarified in this study, the modelling output would still be inevitably affected, e.g. in Italy, where occurrence records are comparably sparse.

In addition, USUV is still spreading in Europe and likely does not occupy its entire environmental niche yet, which may lead to under-estimation of risk through the environmental niche model in areas that may be climatically suitable, but have not been reached yet (compare e.g. [72]). The quality and accessibility of observed records of occurrence of vectors, hosts and especially pathogens is a major practical obstacle for the development of models of the environmental niche model family. Only a consistent and advanced monitoring system covering a selection of representative areas across Europe could give more accurate and reliable occurrence records to produce risk maps. Consequently, the environmental niche model performance can be improved as more occurrence data with high quality are available and the sampling bias is minimized. Ideally, such a monitoring system is centralized, open access and would not only focus on birds or mosquitoes but also include mammalian hosts such as rodents or bats to cover different types of potentially circulating pathogens. Especially the latter have been suspected to be under-estimated but important hosts for other viral zoonotic diseases [73]. As USUV outbreaks typically cease with the arrival of winter, hibernating bats could enable overwintering of the virus. However, coordinated efforts are also needed for centralized and open access to the occurrence records resulting from these improved measures [35].

### Epidemiological model

As an absence of records does not necessarily indicate an absence of risk, it makes sense to use a mechanistic model to point out regions such as southwestern France, where transmission appears to be possible. The SEIR model captured the USUV events in the Pannonian Basin and Po Valley regions well, though the events in Germany and the Netherlands were not represented correctly. Hence, it must be questioned whether the current knowledge on processes, mechanisms and underlying parameters is sufficient to explain USUV transmission patterns and outbreaks. Although an extensive literature review was conducted with the aim of improving and updating the parameters for the SEIR model, no information supporting the integration of additional processes, drivers or variables was found. Therefore, all the parameters and variables used already in the 2008 study of Rubel et al. [44] were kept unchanged, even though some of them are probably not suitable for the whole study area. For instance, population density as well as birth and mortality rates of common blackbirds are unlikely to be constant across the whole study area. An advanced, open-access monitoring system as discussed above could also be of great use for this.

Furthermore, although precipitation is known to affect mosquito life cycles and disease transmission dynamics [74, 75], the applied SEIR model does not take this into account. The SEIR model for USUV was originally developed and calibrated for temperate climates. It is thus possible that certain ecological factors (e.g. precipitation), which are not limiting in the calibration area but could be limiting elsewhere, are not included in the model. In our study we restrained the extent for the SEIR model by excluding climate types with dry seasons in order to avoid making predictions for regions the model is not suitable for. Future models should aim to improve the population model components for vectors and hosts, leading to a more universally useful model. In addition, explicit parameters for USUV are not available yet and had to be substituted by data for the related WNV. For instance, no information about the extrinsic incubation period and its relation to ambient temperature is currently available. Data from a single experiment on a single strain of another virus (i.e. West-Nile virus) [76] is far from optimal, as it has been shown that these experiments are subject to large uncertainty for various reasons [77]. This is a common problem, though, since updated and realistic experiments are sorely needed for many VBDs [35]. Future models could account for some of this uncertainty by incorporating stochastic variations instead of relying on fixed values, as it has already been done e.g. for Chikungunya [78].

Another point worth considering is that so far there is no standardized way of converting the daily values of R_0_ calculated by the SEIR model for each grid cell into interpretable maps. Obviously, some amount of temporal aggregation needs to be applied in order to gain low dimensional, printable maps. In practice, this ranges from R_0_ being displayed as averages for single months (e.g. [79]) up to R_0_ values being averaged over 30-year periods (e.g. [80]). Here, we chose to display average R_0_ values for single transmission seasons, which apparently failed to predict the 2016 USUV event in Northwest Europe (Fig. 4c). However, R_0_ is a threshold value. Thus, while a value of R_0_ > 1 indicates high risk of disease spread, an average R_0_ < 1 for the same period does not necessarily mean no or even low risk, depending on how the length of that period was chosen and how often the threshold was exceeded. This is a serious drawback of SEIR model results to visualize the spatial-explicit risk of pathogen transmission. Hence, an alternative way of illustrating these models is concentrating on the duration of time where R_0_ > 1. Here, we chose to count the (average) number of days per year where R_0_ > 1, but this can also be done on other temporal scales (e.g. months [81]). In our case, this value apparently fails to capture the outbreak area in Germany and the Netherlands (Fig. 2b). However, a closer look reveals that this again is a lack of knowledge about the details of the disease that prevents a meaningful interpretation of these maps, i.e., how many days of R_0_ > 1 are actually needed for an USUV event to occur. When this threshold would be known, the average yearly number of days of R_0_ > 1 map can be converted to a categorized risk map showing whether there is a risk and how severe it is. Furthermore, it has to be questioned, if higher absolute R_0_ values during the transmission season would reduce the number of days of R_0_ > 1 days required for an USUV outbreak. Only when these primary questions are addressed, a more reasonable risk map can be generated.

### Outlook

Further efforts should strive towards the unification of the two streams of modeling. As shown in this study, the ecological niche model reflects spatial distribution better, while the epidemiological model has the advantage of capturing short term variabilities, as it uses daily temperature data. Ecological niche models are run with climate data which typically covers decades, and as a consequence, extreme weather events such as heat waves would not be captured. An integrated model could benefit from both models’ advantages. For example, in a hierarchical approach, spatial distribution of risk could first be estimated by an environmental niche model, followed by a zoom into a finer scale for the investigation of temporal risk patterns in high risk areas through an epidemiological model with well-updated parameters and variables. In this case, the finer temporal scale epidemiological model, using daily weather data or even weather forecast data, can work as a live early warning forecast. Instead of projecting where climate is suitable, ecological niche models can also be applied to exclude unsuitable regions. In addition, in an integrated approach, environmental niche models that estimate the abundance of vectors and hosts could be nested in an epidemiological model as well, in order to gain more precise information on the required vector-to-host ratio.

## Conclusion

In conclusion, this study highlights the necessity to consider different approaches to detect the current and future areas under risk of VBDs. Environmental niche models and epidemiological models examine rather complementary aspects, especially in terms of short-term weather conditions versus long-term climatic conditions. Environmental niche models are typically built upon long-term climate data and thus can be used to gain a general overview of the areas at risk and estimate potential effects of climate change. Given enough spatially explicit occurrence records are available, these models are particularly useful for a rapid risk assessment of emerging VBDs, while more detailed data about the transmission mechanisms is gathered. Once this data is available, elaborate mechanistic models can offer more fine-grained insights on the progression of outbreaks, with the potential for short-term forecasts based on weather models. At this point, environmental niche models for host or vector populations can provide valuable input data for advanced epidemiological models. Thus, using both approaches complementing each other is key for a comprehensive and effective risk evaluation.

Wide parts of Europe are currently at risk of USUV circulation, and its status of a mostly neglected emerging disease makes estimation of its potential future range difficult. Evidence suggests that USUV event s may be more likely to occur in climatically favored regions within Europe such as the Po Valley in northern Italy [82] and the Rhine Valley [48, 50]. At the same time, these areas have a high human population density and exhibit large urban areas and cities. Remnant wetland habitats along rivers serve as habitats for migratory bird stops resulting in a combined setting with humans being exposed to high risk. The detected spatial patterns can be used to indicate regions where surveillance activities should be focused and intensified.

## Additional files

**Additional file 1.** Records of USUV-infected bird locations confirmed by RT PCR collected from the literature.

**Additional file 2.** Buffer radii versus model performance.

**Additional file 3.** Detailed description of the SEIR model.
