# Supplementary material for: Evaluating the risk for Usutu virus circulation in Europe: comparison of environmental niche models and epidemiological models
Source: Int J Health Geogr. 2018 Oct 12;17:35. doi: 10.1186/s12942-018-0155-7 (PMC6186058; doi:10.1186/s12942-018-0155-7)
Supplement: Supplementary file 1 — Additional file 1. Records of USUV-infected bird locations confirmed by RT PCR collected from the literature. [file 12942_2018_155_MOESM1_ESM.docx]

**Additional File 1. Records of USUV-infected bird locations confirmed by PCR collected from the literature.**

| Countries | Outbreak years | Data type | Reference |
| --- | --- | --- | --- |
| Austria | 2003–2005 | Map | [1] |
| Hungary | 2005–2006 | Map | [2] |
| Italy | 2009 | Map | [3] |
| Italy | 2009 | Map | [4] |
| Italy | 2010 | Map | [5] |
| Austria and Hungary | 2010–2016 | Coordinates | [6] |
| Italy | 2011 | Map | [7] |
| Germany | 2011, 2015 | Site description | [8] |
| Czech Republic | 2011–2012 | Coordinates | [9] |
| Germany | 2011–2013 | Map | [10] |
| Belgium | 2012 | Coordinates | [11] |
| Italy | 2012 | Site description | [12] |
| Germany | 2013 | Coordinates | [13] |
| Italy | 2013 | Map | [14] |
| France | 2015 | Site description | [15] |
| Netherlands | 2016 | Map | [16] |

**References**

1. Chvala S, Bakonyi T, Bukovsky C, Meister T, Brugger K, Rubel F, et al. Monitoring of Usutu virus activity and spread by using dead bird surveillance in Austria, 2003-2005. Vet Microbiol. 2007;122(3-4): 237-245. doi: 10.1016/j.vetmic.2007.01.029.

2. Bakonyi T, Erdélyi K, Ursu K, Ferenczi E, Csörgo T, Lussy H, et al. Emergence of Usutu virus in Hungary. J Clin Microbiol. 2007;45(12): 3870-3874. doi: 10.1128/jcm.01390-07.

3. Calzolari M, Bonilauri P, Bellini R, Albieri A, Defilippo F, Maioli G, et al. Evidence of simultaneous circulation of West Nile and Usutu viruses in mosquitoes sampled in Emilia-Romagna region (Italy) in 2009. PLoS One. 2010;5(12): e14324. doi: 10.1371/journal.pone.0014324.

4. Tamba M, Bonilauri P, Bellini R, Calzolari M, Albieri A, Sambri V, et al. Detection of Usutu virus within a West Nile virus surveillance program in northern Italy. Vector Borne Zoonotic Dis. 2011;11(5): 551-557. doi: 10.1089/vbz.2010.0055.

5. Calzolari M, Gaibani P, Bellini R, Defilippo F, Pierro A, Albieri A, et al. Mosquito, bird and human surveillance of West Nile and Usutu viruses in Emilia-Romagna region (Italy) in 2010. PLoS One. 2012;7(5): e38058. doi: 10.1371/journal.pone.0038058.

6. Bakonyi T, Erdélyi K, Brunthaler R, Dán Á, Weissenböck H, Nowotny N. Usutu virus, Austria and Hungary, 2010-2016. Emerg Microbes Infect. 2017;6(10): e85. doi: 10.1038/emi.2017.72.

7. Calzolari M, Bonilauri P, Bellini R, Albieri A, Defilippo F, Tamba M, et al. Usutu virus persistence and West Nile virus inactivity in the Emilia-Romagna region (Italy) in 2011. PLoS One. 2013;8(5): e63978. doi: 10.1371/journal.pone.0063978.

8. Ziegler U, Fast C, Eiden M, Bock S, Schulze C, Hoeper D, et al. Evidence for an independent third Usutu virus introduction into Germany. Vet Microbiol. 2016;192: 60-66. doi: 10.1016/j.vetmic.2016.06.007.

9. Hubálek Z, Rudolf I, Čapek M, Bakonyi T, Betášová L, Nowotny N. Usutu virus in blackbirds (*Turdus merula*), Czech Republic, 2011-2012. Transbound Emerg Dis. 2014;61(3): 273-276. doi: 10.1111/tbed.12025.

10. Ziegler U, Jöst H, Müller K, Fischer D, Rinder M, Tietze DT, et al. Epidemic spread of Usutu virus in southwest Germany in 2011 to 2013 and monitoring of wild birds for Usutu and West Nile viruses. Vector Borne Zoonotic Dis. 2015;15(8): 481-488. doi: 10.1089/vbz.2014.1746.

11. Garigliany MM, Marlier D, Tenner-Racz K, Eiden M, Cassart D, Gandar F, et al. Detection of Usutu virus in a bullfinch (*Pyrrhula pyrrhula*) and a great spotted woodpecker (*Dendrocopos major*) in north-west Europe. Vet J. 2014;199(1): 191-193. doi: 10.1016/j.tvjl.2013.10.017.

12. Grisenti M, Vázquez A, Herrero L, Cuevas L, Perez-Pastrana E, Arnoldi D, et al. Wide detection of Aedes flavivirus in north-eastern Italy - a European hotspot of emerging mosquito-borne diseases. J Gen Virol. 2015;96: 420-430. doi: 10.1099/vir.0.069625-0.

13. Cadar D, Becker N, Campos RDM, Börstler J, Jöst H, Schmidt-Chanasit J. Usutu virus in bats, Germany, 2013. Emerg Infect Dis. 2014;20(10): 1771-1773. doi: 10.3201/eid2010.140909.

14. Calzolari M, Pautasso A, Montarsi F, Albieri A, Bellini R, Bonilauri P, et al. West Nile virus surveillance in 2013 via mosquito screening in northern Italy and the influence of weather on virus circulation. PLoS One. 2015;10(10): e0140915. doi: 10.1371/journal.pone.0140915.

15. Lecollinet S, Blanchard Y, Manson C, Lowenski S, Laloy E, Quenault H, et al. Dual emergence of Usutu virus in common blackbirds, eastern France, 2015. Emerg Infect Dis. 2016;22(12): 2225-2227. doi: 10.3201/eid2212.161272.

16. Rijks J, Kik M, Slaterus R, Foppen R, Stroo A, Ijzer J, et al. Widespread Usutu virus outbreak in birds in the Netherlands, 2016. Euro Surveill. 2016;21(45): pii=30391. doi: 10.2807/1560-7917.es.2016.21.45.30391.
