# Supplementary figures and images for: Evaluating the risk for Usutu virus circulation in Europe: comparison of environmental niche models and epidemiological models
Source: Int J Health Geogr. 2018 Oct 12;17:35. doi: 10.1186/s12942-018-0155-7 (PMC6186058; doi:10.1186/s12942-018-0155-7)

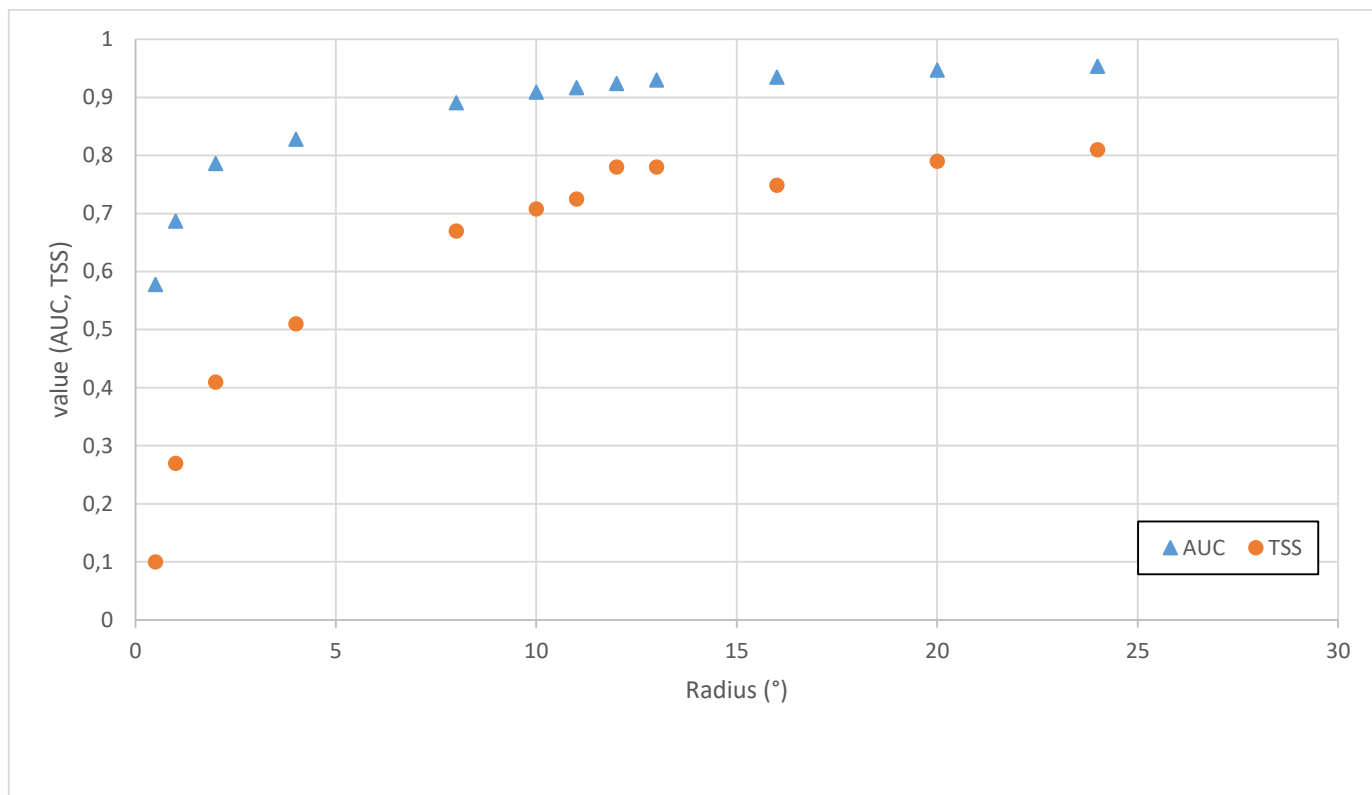

Supplement: Supplementary file 2 — Additional file 2. Buffer radii versus model performance. [file 12942_2018_155_MOESM2_ESM.pdf]
