# Supplementary material for: Evaluating the risk for Usutu virus circulation in Europe: comparison of environmental niche models and epidemiological models
Source: Int J Health Geogr. 2018 Oct 12;17:35. doi: 10.1186/s12942-018-0155-7 (PMC6186058; doi:10.1186/s12942-018-0155-7)
Supplement: Supplementary file 3 — Additional file 3. Detailed description of the SEIR model. [file 12942_2018_155_MOESM3_ESM.docx]

A simplified diagram chart of Usutu virus (USUV) epidemiological model:


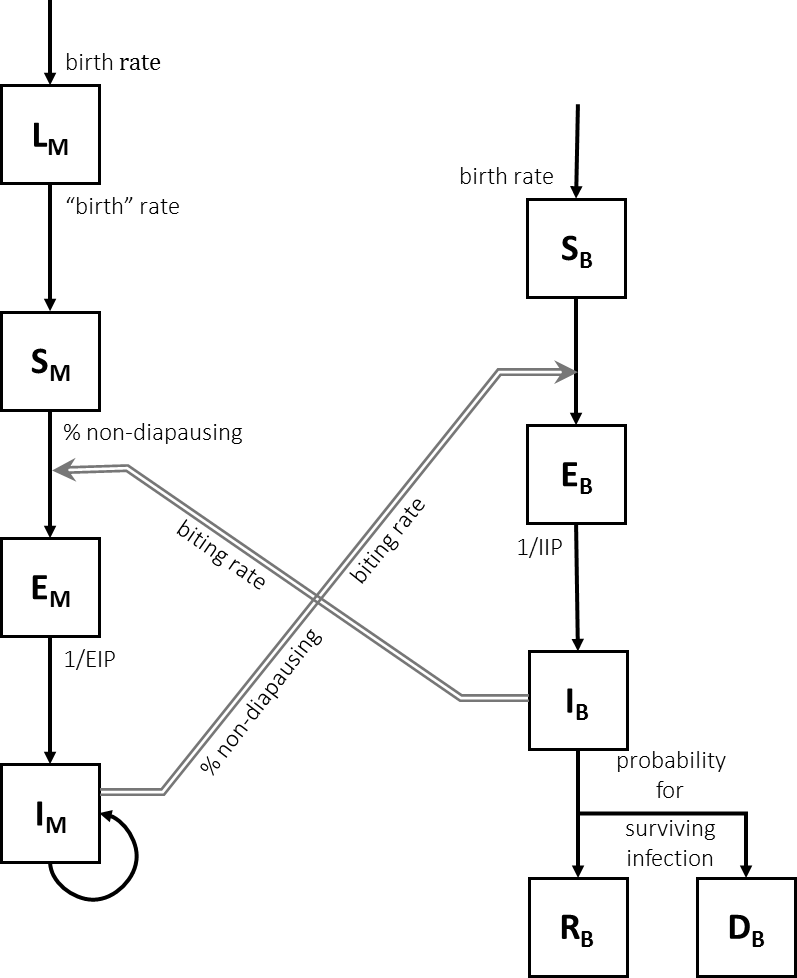


Each health state of mosquitoes / black birds can be described by Ordinary differential equations (ODEs).

Population growth of black birds:

$$\frac{dN_{B}}{dt}=r_{B}N_{B}=b_{B}N_{B}-m_{B}N_{B}$$

$N_{B}$ is the total number of black birds, $r_{B}$ is the population growth rate, $b_{B}$is the birth rate and $m_{B}$ is the mortality rate (B stands for black birds).

Follow logistic population growth (density dependent model):

$$\frac{dN_{B}}{dt}=r_{B}\left( 1-\frac{N_{B}}{K_{B}} \right)N_{B}$$

As $r_{B}=b_{B}-m_{B}$

it can be written as:

$$\frac{dN_{B}}{dt}=\left( b_{B}-\left( b_{B}-m_{B} \right)\frac{N_{B}}{K_{B}} \right)N_{B}-m_{B}N_{B}$$

$K_{B}$ stands for environmental capacity. It can be understood as the maximum number of individuals that can be supported by the environment under ideal conditions.

The population of “larval” mosquitoes (includes all aquatic stages of *Culex* mosquitoes, only females taken into account) also follows logistic population growth:

$$\frac{dL_{M}}{dt}=\left( b_{L}N_{M}-m_{L}L_{M} \right)\left( 1-\frac{L_{M}}{K_{M}} \right)-b_{M}L_{M}$$

$L_{M}$ is the total number of larvae, $b_{L}$ is the birth rate of larvae, $m_{L}$ is the mortality rate of larvae, $b_{M}$ is the “birth rate” of mosquitoes (transformation from larvae to adult mosquitoes). Note here: although also following logistic population growth, mosquito growth is divided to aquatic and terrestrial stages, thus the equation looks different from black birds’.

Total density of terrestrial stages of *Culex* mosquitoes ($N_{M}$):

$$\frac{dN_{M}}{dt}=b_{M}L_{M}-m_{M}N_{M}$$

Cross-infection between mosquitoes and black birds:

$$\lambda_{B}=\beta_{B}\frac{I_{B}}{K_{B}}=\kappa P_{B}\frac{I_{B}}{K_{B}}$$

$$\lambda_{M}=\beta_{M}\frac{I_{M}}{K_{B}}=\kappa P_{M}\frac{I_{M}}{K_{B}}$$

$\lambda_{B}$ denote the possible fraction of cross-transmission from birds to mosquitoes, and $\lambda_{M}$ vice versa. $\beta_{B}$ is the product of biting rate ($\kappa$) and transmission possibility from birds to mosquitoes($P_{B}$), and $\beta_{M}$ vice versa. Transmission possibility from mosquitoes to birds is $P_{M}$.

Then the different health states of birds can be described by following ODEs:

1. The susceptible black bird population ($S_{B}$)

$$\frac{dS_{B}}{dt}=\left( b_{B}-\left( b_{B}-m_{B} \right)\frac{N_{B}}{K_{B}} \right)N_{B}-m_{B}N_{B}-\delta_{M}\lambda_{M}S_{B}$$

It can be understood as:

(current total number of susceptible black birds) = (current total number birds) $-$ (natural death of birds) $-$ (birds moving to the next health state)

Here “natural death of birds” means deaths not due to Usutu virus (USUV) infection.

1. The exposed black bird population ($E_{B}$)

$$\frac{dE_{B}}{dt}=\delta_{M}\lambda_{M}S_{B}-m_{B}E_{B}-\gamma_{B}E_{B}$$

$\delta_{M}$ : Percentage of non-hibernating mosquitoes

$\gamma_{B}$ : The exposed – infected/infectious rate of birds

From this equation:

(current total number of exposed black birds) = (birds coming into this health state from the previous stage) $-$ (natural death of birds) $-$ (birds moving to the next health state)

1. The infected black bird population ($I_{B}$)

$$\frac{dI_{B}}{dt}=\gamma_{B}E_{B}-m_{B}I_{B}-\alpha_{B}I_{B}$$

$\alpha_{B}$: The removal rate, removed from the previous health state, either get recovered (immunized) or dead

Similar as 2.

1. The black bird deaths ($D_{B}$) due to USUV infection

$$\frac{dD_{B}}{dt}={\nu_{B}\alpha}_{B}I_{B}$$

$\nu_{B}$: the percentage of bird deaths due to USUV infection

1. The recovered black bird population ($R_{B}$)

$$\frac{dR_{B}}{dt}=\left( 1-\nu_{B} \right)\alpha_{B}I_{B}-m_{B}R_{B}$$

And

$N_{B}=S_{B}+E_{B}+I_{B}+R_{B}$

Note: In this model both horizontal and vertical virus transmission in birds are not taken into account, so the transmission is limited to through mosquitoes’ blood meal.

Similarly, the different health states of mosquitoes are described as following:

1. The larval population of *Culex* mosquitoes:

$$\frac{dL_{M}}{dt}=\left( b_{L}N_{M}-m_{L}L_{M} \right)\left( 1-\frac{L_{M}}{K_{M}} \right)-b_{M}L_{M}$$

2. The susceptible mosquito population:

$$\frac{dS_{M}}{dt}=b_{M}L_{M}-m_{M}S_{M}-\delta_{M}\lambda_{B}S_{M}$$

From this equation, similar to bird equations:

(current total number of susceptible mosquitoes) = (mosquitoes entering this health state from the previous stage) – (natural death of mosquitoes) – (mosquitoes moving to the next health state).

1. The exposed mosquito population:

$$\frac{dE_{M}}{dt}=\delta_{M}\lambda_{B}S_{M}-m_{M}E_{M}-\gamma_{M}E_{M}$$

$\gamma_{M}$: The exposed – infected/infectious rate of mosquitoes

1. The infected mosquito population:

$$\frac{dI_{M}}{dt}=\gamma_{M}E_{M}-m_{M}I_{M}$$

And

$$N_{M}=S_{M}+E_{M}+I_{M}$$

Note: Infectious mosquitoes remain in the infectious state and will not get recovered.

In addition, $\delta_{M}$ is determined by the latitude and the calendar day of the year.

$$\delta_{M}=1-\frac{1}{1+1775.7\exp\left[ 1.559\left( D-18.177 \right) \right]}$$

Of which *D* denotes “Daytime length”, and

$$D=7.639arcsin\left[ tan\left( \epsilon\right)tan\left( \varphi\right)+\frac{0.0146}{cos\left( \epsilon\right)cos\left( \varphi\right)} \right]+12$$

$$\epsilon=0.409sin\left( \frac{2\pi\left( d-80 \right)}{365} \right)$$

$\varphi$: Geographic latitude

$d$: The calendar day

The final R_0_ equation:

$$R_{0} = \sqrt{\left[ \frac{\delta_{M}\gamma_{M}\beta_{M}}{\left( \gamma_{M}+m_{M} \right)m_{M}}\frac{S_{B}}{K_{B}} \right]\left[ \frac{\delta_{M}\gamma_{B}\beta_{B}}{\left( \gamma_{B}+m_{B} \right)\left( \alpha_{B}+m_{B} \right)}\frac{S_{M}}{K_{B}} \right]}$$

Additional table.1 Parameters for R_0_ equation

|  | parameter | value |
| --- | --- | --- |
| the population growth rate | $r_{B}$ | $r_{B}=b_{B}-m_{B}$ |
| birth rate | $b_{B}$ | $b_{B}(d)=0.125\frac{\left( x/\beta\right)^{\alpha-1}exp(-x/\beta)}{\beta\Gamma\left( \alpha\right)}$ |
|  |  | $x=0.1(d-105)$, $d$ is transformed Julian calendar day |
|  |  | $\alpha$=1.52, $\beta$=1.93, $\Gamma\left( \alpha\right)$=0.887 |
| mortality rate | $m_{B}$ | 0.0012 |
| the birth rate of larvae | $b_{L}$ | $b_{L}(T)=2.325\kappa(T)$ |
|  |  | $T$ :Daily Mean Temperature |
| mortality rate of larvae | $m_{L}$ | $m_{L}(T)=0.0025T^{2}-0.094T+1.0257$ |
| “birth rate” of mosquitoes (transformation from larvae to adult mosquitoes). | $b_{M}$ | $b_{M}(T)=0.1b_{L}$ |
| mortality rate of mosquitoes | $m_{M}$ | $m_{M}(T)=0.1m_{L}$ |
| possible fraction of cross-transmission from birds to mosquitoes | $\lambda_{B}$ | $\lambda_{B}(T)=\beta_{B}(T)\frac{I_{B}}{K_{B}}=\kappa(T)P_{B}\frac{I_{B}}{K_{B}}$ |
| product of biting rate ($\kappa$) and transmission possibility from birds to mosquitoes($P_{B}$) | $\beta_{B}$ | $\beta_{B}\left( T \right)=\kappa\left( T \right)P_{B}$ $P_{B}$=$0.125$ |
| biting rate | $\kappa$ | $\kappa(T)=\frac{0.344}{1+1.231exp(-0.184(T-20))}$ |
| possible fraction of cross-transmission from mosquitoes to birds | $\lambda_{M}$ | $\lambda_{M}(T)=\beta_{M}(T)\frac{I_{M}}{K_{B}}=\kappa(T)P_{M}\frac{I_{M}}{K_{B}}$ |
| product of biting rate ($\kappa$) and transmission possibility from mosquitoes to birds ($P_{M}$) | $\beta_{M}$ | $\beta_{M}\left( T \right)=P_{M}\kappa\left( T \right)$ |
| Percentage of non-hibernating mosquitoes | $\delta_{M}$ | $\delta_{M}=1-\frac{1}{1+1775.7\exp\left[ 1.559\left( D-18.177 \right) \right]}$ |
|  |  | $D=7.639arcsin\left[ tan\left( \epsilon\right)tan\left( \varphi\right)+\frac{0.0146}{cos\left( \epsilon\right)cos\left( \varphi\right)} \right]+12$ |
|  |  | $\epsilon=0.409sin\left( \frac{2\pi\left( d-80 \right)}{365} \right)$ |
| exposed – infected/infectious rate of birds | $\gamma_{B}$ | 0.667 |
| removal rate, removed from the previous health state, either get recovered (immunized) or dead | $\alpha_{B}$ | 0.182 |
| the percentage of bird deaths due to USUV infection | $\nu_{B}$ | 0.3 |
| The exposed – infected/infectious rate of mosquitoes | $\gamma_{M}$ | $\gamma_{M}\left( T \right)=0.0093T-0.1352$,  $T\geq{15}^{^{\circ}}$ $\gamma_{M}\left( T \right)=0$, $T<{15}^{^{\circ}}$ |

* Note that highlighted parameters are also documented in Table 2.
